# Unlocking Diagnostic Precision: FATE Protocol Integration with BLUE and eFAST Protocols for Enhanced Pre-Hospital Differential Diagnosis of Pleural Effusion Manifested as Dyspnea in Adults—A Pilot Study

**DOI:** 10.3390/jcm13061573

**Published:** 2024-03-09

**Authors:** Damian Kowalczyk, Miłosz Turkowiak, Wojciech Jerzy Piotrowski, Oskar Rosiak, Adam Jerzy Białas

**Affiliations:** 1 Department of Pneumology, 2nd Chair of Internal Medicine, Medical University of Lodz, 90-153 Lodz, Poland; damiankowalczyk@onet.pl (D.K.); wojciech.piotrowski@umed.lodz.pl (W.J.P.); 2 Department of Anesthesiology and Intensive Care, National Institute of Medicine, Ministry of the Interior and Administration, 02-507 Warsaw, Poland; milosztr@gmail.com; 3 Department of Otolaryngology, Polish Mother’s Memorial Hospital Research Institute, 93-338 Lodz, Poland; oskar.rosiak@iczmp.edu.pl; 4 Department of Pulmonary Rehabilitation, Regional Medical Center for Lung Diseases and Rehabilitation, Blessed Rafal Chylinski Memorial Hospital for Lung Diseases, 91-520 Lodz, Poland

**Keywords:** pre-hospital assessment, pleural effusion, thoracic ultrasound, point-of-care ultrasound, POCUS, focus assessed transthoracic echocardiography, FATE, BLUE, eFAST

## Abstract

**Background**: Dyspnea commonly stems from combined myocardial and pulmonary dysfunction, posing challenges for accurate pre-hospital diagnosis. Limited diagnostic capabilities hinder the differentiation of cardiac and pulmonary issues. This study assesses the efficacy of combined cardiac and pulmonary ultrasound using the BLUE, eFAST, and FATE protocols. **Methods**: Participants were consecutively enrolled from dyspnea-related emergency calls in Warsaw, Poland, from 4 April 2022, to 15 June 2023. Patients with pleural effusion were identified through pre-hospital and in-hospital radiological assessments. Pre-hospital thoracic ultrasonography followed the BLUE, eFAST, and FATE protocols, alongside comprehensive clinical assessments. The pre-hospital diagnoses were juxtaposed with the with hospital discharge diagnoses. **Results**: Sixteen patients (8 men, 8 women; median age: 76 years) were enrolled. Inter-rater agreement for the BLUE protocol was substantial (κ = 0.78), as was agreement for eFAST (κ = 0.75), with almost perfect agreement for combined protocol assessment (κ = 0.83). Left ventricle hypokinesis, identified via the FATE protocol, significantly correlated with hospital-diagnosed decompensated heart failure as the primary cause of dyspnea. Sensitivity and specificity were 1.0 (95%CI: 0.62–1.0) and 0.6 (95%CI: 0.15–0.95), respectively. Positive predictive value was 0.85 (95%CI: 0.55–0.98), and diagnostic accuracy was 0.86 (95%CI: 0.62–0.98). **Conclusions**: Integrating the FATE protocol into BLUE and eFAST enhances pre-hospital differential diagnosis accuracy of pleural effusion in adults. This synergistic approach streamlines diagnostic processes and facilitates informed clinical decision-making. Larger-scale validation studies are needed for broader applicability.

## 1. Introduction

**Figure 1:**
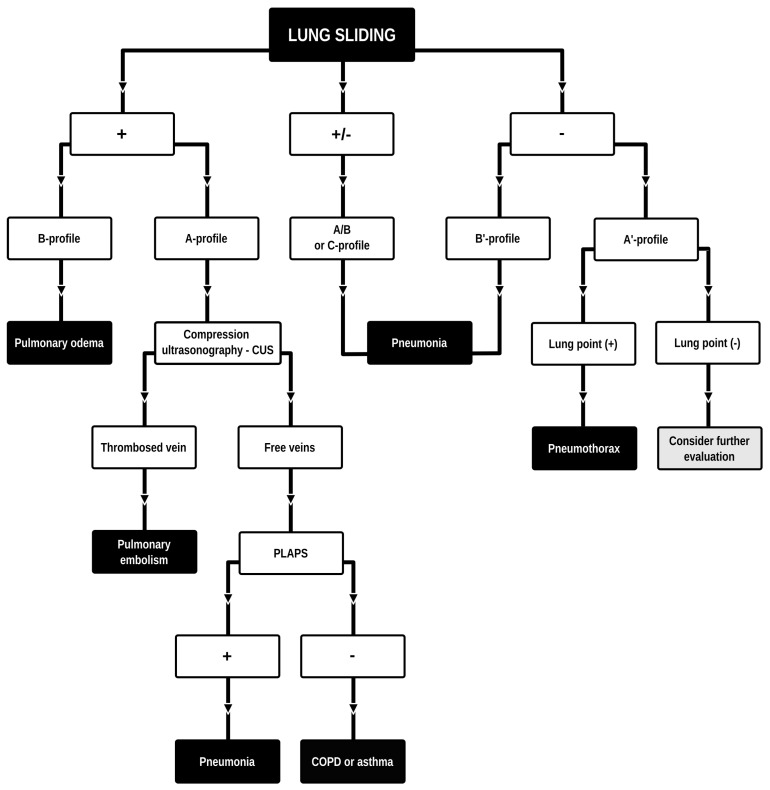
BLUE (Bedside Lung Ultrasound in Emergency) protocol diagram. Adapted and modified from: Lichtenstein et al. [6]. Lung ultrasound examination protocol focused on the differential diagnosis of pulmonary edema, pneumothorax, COPD, pulmonary embolism, or pneumonia.

**Figure 2:**
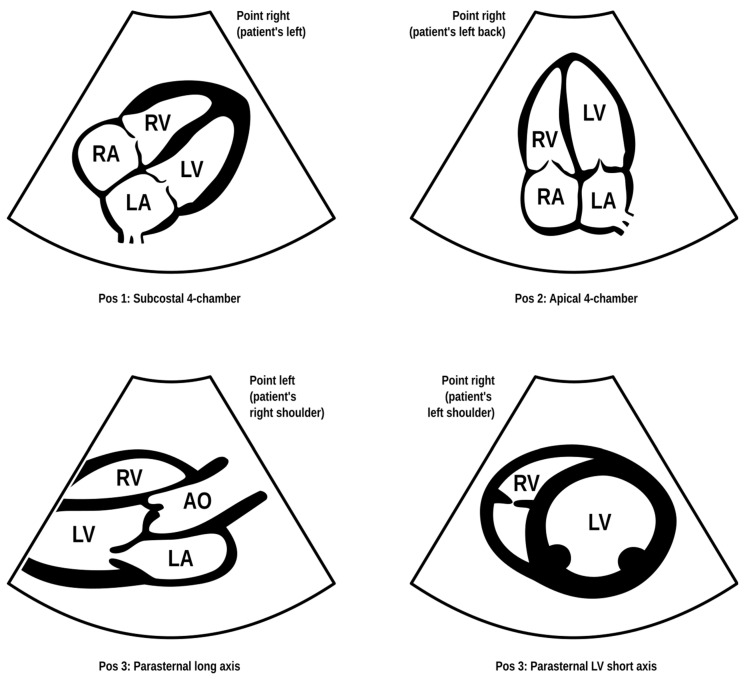
FATE (Focus Assessed Transthoracic Echo) protocol. Modified based on the FATE protocol by Erik Sloth—FATE card. Cardiac ultrasound examination protocol in terms of basic echo, cardiac imaging in four projections enabling the identification of, among others, cardiac tamponade, right ventricular overload, or severe left ventricular hypokinesis (source: author’s material—DK).

**Figure 3:**
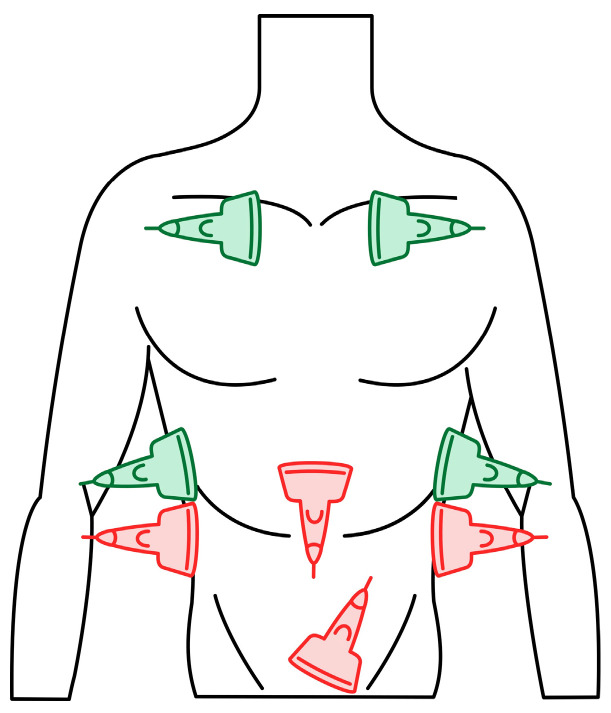
eFAST (Extended Focused Assessment with Sonography for Trauma) protocol. Trauma ultrasound examination protocol describing basic imaging diagnostics of the abdominal cavity and chest. The traditional FAST protocol (probes marked in red) was supplemented with an examination of lung fields (probes marked in green) (source: author’s material—DK).

Dyspnea and pleural effusion are common signs of decompensated heart failure [1,2]. Thoracic ultrasonography seems to have a high incidence of detecting pleural effusion and has demonstrated a high predictive accuracy for identifying patients with decompensated heart failure, while the incidence of physical and radiographic signs of pleural effusion is relatively low [2,3,4]. Furthermore, distinguishing between decompensated heart failure and respiratory diseases, such as pneumonia or chronic obstructive pulmonary disease can be challenging, given their overlapping demographics and coexistence. Consequently, pre-hospital chest sonography is considered a valuable adjunct to history and clinical examination findings. The authors of this study identified lung ultrasound as a useful diagnostic tool for pre-hospital differential diagnosis of dyspnea in adults. Building upon observations from our previous study [5], it is plausible that augmenting widely used POCUS (point-of-care ultrasound) protocols such as the BLUE (Bedside Lung Ultrasound in Emergency). The BLUE protocol, which is specific for ultrasound diagnosis of lung diseases, is performed by examining a minimum of six sites on the chest (three evaluations on each side of the chest); depending on the patient’s condition, this examination is performed on the anterior side of the chest—patients lying down—or from the posterior side of the chest in patients able to sit up during the LUS (lung ultrasound) examination. The sites of ultrasound probe application are the area of the II-III intercostal space, VI-VIII intercostal space, and the area of the rib-pericardium/PLAPS (posterolateral alveolar and/or pleural syndrome)—basally in the area of the posterior axillary line. In the LUS examination according to the BLUE protocol, sonographic changes are evaluated according to a pleural-line sliding motion, A-line (A profile), B-line (B profile), and C-line (C profile) artifacts, subpleural consolidations, the presence of a lung point, and a deep vein assessment on ultrasound—a femoral and popliteal vein compression test. According to the BLUE protocol, the following are diagnosed and differentiated: pulmonary embolism, exacerbation of obstructive diseases, pneumothorax, and stasis in the pulmonary circulation, among others (Figure 1). Utilizing this protocol for lung examinations, the investigator places the ultrasound probe on both sides of the chest in at least three places—at the top of the chest, in the middle, and at the base. Patients are most often examined in a sitting position, assessing the lungs from the back. The second LUS examination protocol in our study is a lung examination according to the eFAST (Extended Focused Assessment with Sonography for Trauma) protocol. This examination, normally used in trauma patients, assists investigators in the differential diagnosis of pneumothorax and traumatic thoracic hemorrhage (hemothorax). The eFAST protocol images the pulmonary fields at four sites, most typically in patients lying down (two on each side of the chest), with ultrasound probe applications at the top of the thorax (the region of the II-III intercostal space in the midclavicular line) and parasternal in the region of the retromediastinum. Patients with symptoms of respiratory failure without a history of trauma are also subjected to the examination in this protocol. An integral part of the examination of the patients described in this article is the ultrasound diagnosis of the heart, following the FATE (Focus Assessed Transthoracic Echo) protocol (Figure 2). This examination is performed in four projections: subcostal long-axis projection, parasternal long-axis projection, parasternal short-axis projection, and apical projection. Based on this information the following diagnoses are made: pericardial effusion, right ventricular overload, hypokinesis, signs of dehydration, dilatation of the ascending aorta, or visual oscillation of the cardiac ejection fraction. The last protocol used in our study is the eFAST ultrasound protocol, which interestingly, includes both cardiac diagnostics (substernal application in the long axis of the heart) according to the FAST protocol. Additionally, in this protocol, the lungs are assessed by scanning the chest in two places (at the top and the base) on both sides of the chest (Figure 3).

A common denominator of our work is the combination of heart and lung diagnostics performed through ultrasound examinations in patients with respiratory dysfunction. Many times, patients (especially in the adult population) with multiple comorbidities are treated for heart and lung diseases, which alone or combined may cause shortness of breath requiring pre-hospital treatment. The correlation of these two organs (heart and lungs) also requires their joint diagnosis during the treatment of respiratory failure. Therefore, in the cohort included in our study, we proposed a combination of the BLUE, FATE, and eFAST protocols to comprehensively diagnose the problem of shortness of breath in pre-hospital conditions. Focusing on feasibility, we selected a population of patients presenting with both dyspnea and pleural effusion. The aim was to assess the diagnostic accuracy of the integration of the FATE protocol with the BLUE and eFAST ones for the pre-hospital differential diagnosis of pleural effusion in adults manifesting as dyspnea.

## 2. Materials and Methods

### 2.1. Study Participants

Similarly to our previous study [5], participants were consecutively recruited from dyspnea-related emergency calls in the Warsaw region of Poland. All enrolled patients were admitted to the emergency department at Priest Jerzy Popieluszko Memorial Hospital in Warsaw. The enrollment period spanned from 4 April 2022 to 15 June 2023. For the analysis, we identified patients with pleural effusion through radiological assessments conducted both in the pre-hospital and in-hospital settings.

The sole exclusion criterion was the inability to provide informed consent for study participation.

Informed consent was obtained from all subjects involved in the study. The study protocol was approved by the Ethics Committee of the Medical University of Lodz, dated 12 April 2022 (protocol No. RNN/69/22/KE).

### 2.2. Data Collection

Data on demographics and clinical characteristics were collected.

In pre-hospital settings, thoracic ultrasonography was conducted following the BLUE [6] as well as the eFAST protocols [7], with the additional incorporation of the FATE protocol [8]. The examinations in the clinical pre-hospital setting were performed by one of the investigators (DK), a paramedic with several years of experience in performing POCUS/LUS examinations confirmed by numerous certifications in ultrasound diagnostics including ECHO and LUS. Subsequently, all the ultrasound projections/images performed were evaluated and described by two more authors of the paper (AB) and (MT). The authors, as POCUS practicing physicians, had clinical experience in the differential diagnosis of patients with respiratory failure gained in the departments of pulmonology (AB) and anesthesiology and intensive care (MT). Standardization of ultrasound examinations has been developed, which means that all examinations in given protocols were performed in the same way for each patient. This applied to both heart and lung diagnostics. This allowed researchers to create uniform ultrasound descriptions, which were then compared with diagnostic imaging performed in hospital conditions, e.g., chest computed tomography.

Exclusion criteria in the study encompassed impaired consciousness, operationalized by a Glasgow Coma Scale (GCS) score falling below 10 points. Consequently, individuals who were unconscious and unable to provide informed consent for participation were excluded from the study. The study also did not include patients under 18 years of age and adolescents. The study included all adult patients with symptoms of respiratory failure who reported shortness of breath and during physical examination presented an accelerated respiratory rate (above 20 breaths per minute) and a saturation value without oxygen supplementation below 90%.

**Figure 4:**
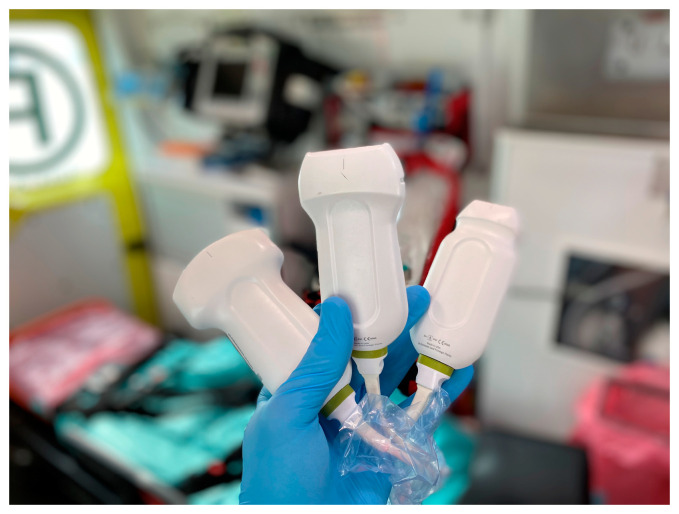
USG probes, from the left: convex probe, linear probe, and sector probe (source: author’s material—DK).

**Figure 5:**
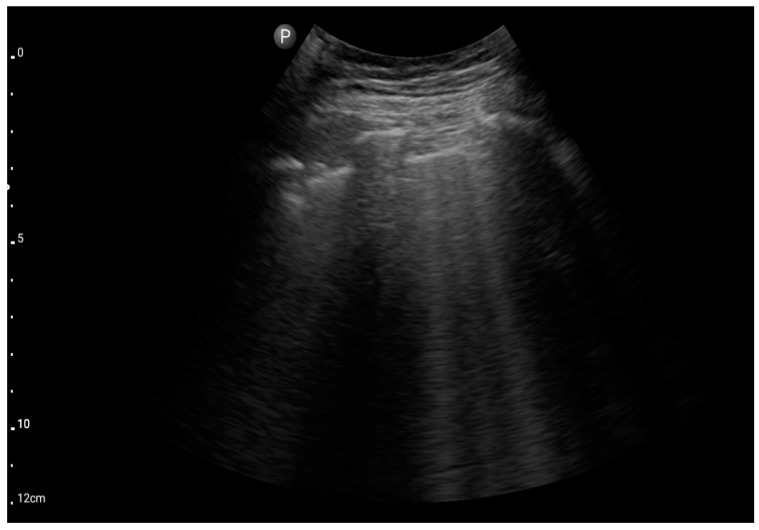
Convex probe; view: lungs, two intercostal spaces-“Merlin space” with B-profile (BLUE protocol), (source: author’s material—DK).

**Figure 6:**
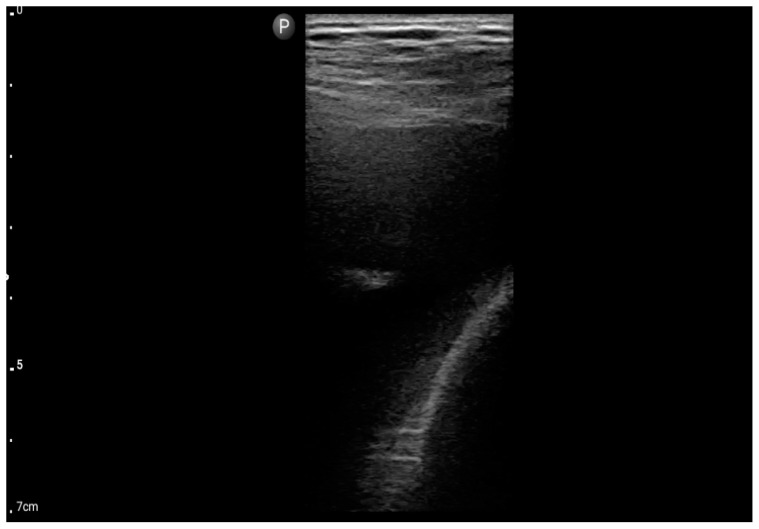
Linear probe; view: Costodiaphragmatic recess—pleural effusion (BLUE protocol) (source: author’s material—DK).

We used three ultrasonographic probes (Figure 3). The lungs were visualized using two ultrasonographic probes: a convex probe (2–5 MHz, 67.3% field of view, 50 mm scanning plane), (Figure 4), and a linear probe (4–12 MHz, 34.5% field of view, 34 mm scanning plane), (Figure 5). The heart was visualized using a sector probe (1–4 MHz, 90% field of view, 50 mm scanning plane), (Figure 6). The examination was performed using a Philips Lumify ultrasound device, Philips Ultrasound LLC, Bothell, DC, USA, 2021, using the probes described above. The BLUE and eFAST protocols were used as described in our previous study [5]. Examination in the BLUE protocol was based on the assessment of the lungs on both sides of the chest, with the assumption of using a minimum of three applications of the ultrasound probe from the front and the back (a total of at least six applications of the ultrasound probe: in the upper, middle, and lower part of the chest). The chest was examined from the sides, but in many patients who were unable to lie down, only the back was examined in a sitting position. The eFAST protocol was most often used in trauma patients but also in patients with heart and lung diseases who were chronically bedridden and unable to sit. In addition to imaging the abdomen, a chest examination was performed in this protocol, including scanning the lungs on both sides—at the base and the top of the lungs. The most frequently differentiated disease entities were pleural effusion and pneumothorax. Following the FATE protocol, we used the sector ultrasound probe in four positions: subcostal four-chamber view, apical four-chamber view, parasternal long axis, and left ventricle short axis, as described by Frederiksen et al. [8]. Pleural scanning was omitted due to the prior completion of BLUE and eFAST assessments. For the sake of systematicity and image comparability, all ultrasound examinations were conducted using the same ultrasound machine, the Philips Lumify system.

In addition to the aforementioned POCUS (based on rapid bedside ultrasound diagnostics, e.g., FATE or BLUE) protocols, we conducted a comprehensive clinical assessment, encompassing medical history, complete physical examination, and fundamental emergency diagnostics (electrocardiography, pulse oximetry, capnometry). Each initial diagnosis was consistently documented in the medical rescue actions card, a component of the patient’s official medical record. This card served as the basis for comparing the pre-hospital diagnosis with the final hospital-acquired diagnosis.

The pre-hospital diagnosis was juxtaposed with the ultimate diagnosis determined on the day of hospital discharge. Consequently, our study represents a real-life evaluation of these POCUS protocols.

### 2.3. Statistical Analysis

Age was reported as the median and interquartile range (IQR) from the 25th to the 75th percentile due to non-normal distribution, as determined by the Shapiro–Wilk test. Categorical data were reported as absolute values and percentages, with comparisons conducted using Fisher’s exact test. Selected parameters were expressed as sensitivity and specificity, positive predictive value, as well as diagnostic accuracy, along with corresponding 95% confidence intervals (CI). Inter-rater agreement was assessed using the Fleiss kappa for nominal values and multiple raters.

The analysis was conducted using R software version 4.3.1.

## 3. Results

### 3.1. Participants’ Characteristics

We enrolled 16 patients in the study—8 (50%) men and 8 (50%) women with a median age of 76 [IQR: 72.5–85.25] years. Nine patients were excluded from the initial twenty-five patients due to an exacerbation of obstructive pulmonary disease. No gender predominance was observed based on the final diagnosis (*p* = 0.71). The table describes 18 diagnoses for a group of 16 patients. This is because in two patients, based on a joint cardiac ultrasound examination (FATE) and LUS examination (BLUE), two simultaneous diagnoses requiring treatment were made: exacerbation of heart failure and pneumonia. The baseline characteristics of the patients are presented in Table 1.

### 3.2. The Effectiveness of POCUS in the Pre-Hospital Setting

The POCUS performed by the paramedic was concordant with the discharge diagnosis in 90.91% of the final diagnoses established on the day of discharge from the hospital.

### 3.3. The Assessment According to the BLUE and eFAST Protocols Integrated with the FATE Protocol

The inter-rater agreement was assessed using Fleiss kappa measurement and interpreted by Landis et al. [9]. For the evaluation using the BLUE protocol, the inter-rater agreement between the three independent raters was 0.78, which was interpreted as substantial agreement. For the eFAST evaluation, the Fleiss kappa was 0.75, also indicating substantial agreement. For the assessment with both protocols, the inter-rater agreement was evaluated separately and was 0.83, indicating almost perfect agreement.

Table 2 below shows the ultrasound diagnostic results of 25 patients with respiratory failure in whom initial lung ultrasound diagnostics were performed (BLUE/eFAST protocol), on the basis of which a preliminary diagnosis was made based only on the evaluation of pathologies assessed in LUS. Then, cardiac imaging was performed (FATE protocol), and the diagnosis was made based only on the evaluation of the heart by the ECHO protocol. Finally, both images (BLUE/eFAST and FATE) were compared and correlated into the final diagnosis described in the table. Table 3 compares the diagnosis results of three investigators.

**Figure 7:**
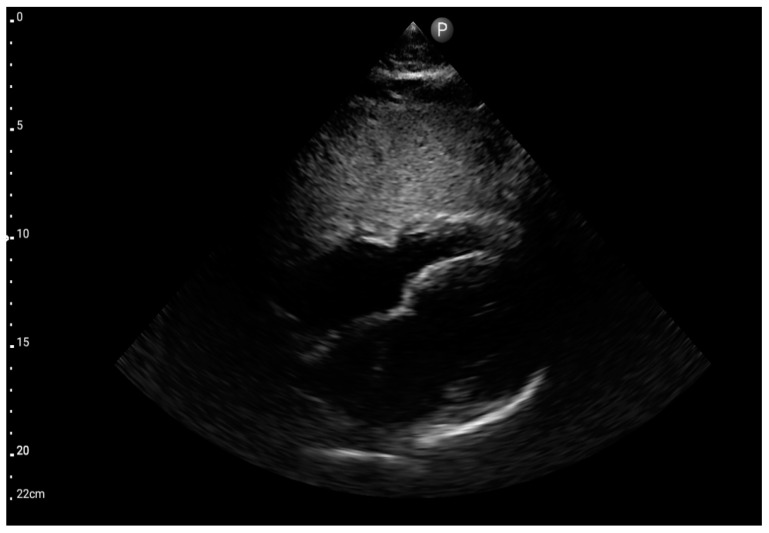
Sector probe; view: subcostal four-chamber (FATE protocol) (source: author’s material—DK).

**Figure 8:**
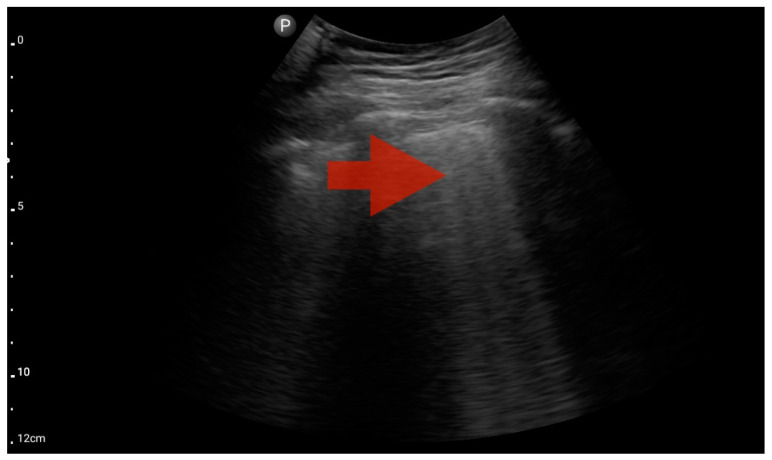
Profile B (BLUE protocol). Convex probe, top of the lung (2nd–3rd intercostal space), the red arrow indicates the vertical artifact of the B line (source: author’s material—DK).

**Figure 9:**
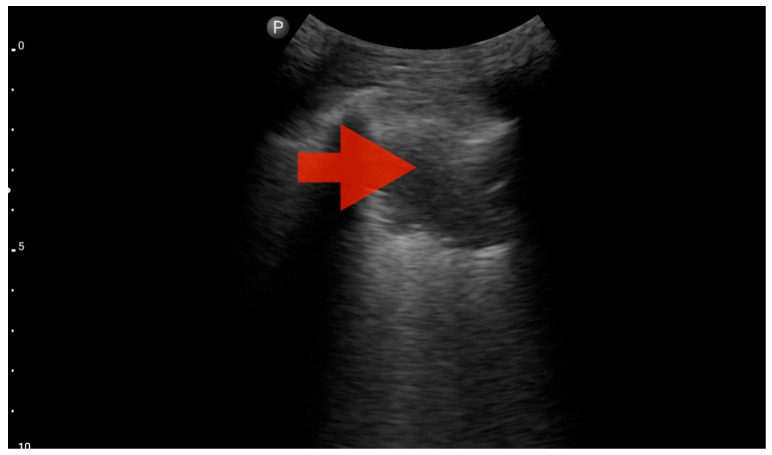
Profile C (BLUE protocol). Convex probe examination, the middle part of the lung (5–7th intercostal); the red arrow indicates subpleural consolidation (source: author’s material—DK).

**Figure 10:**
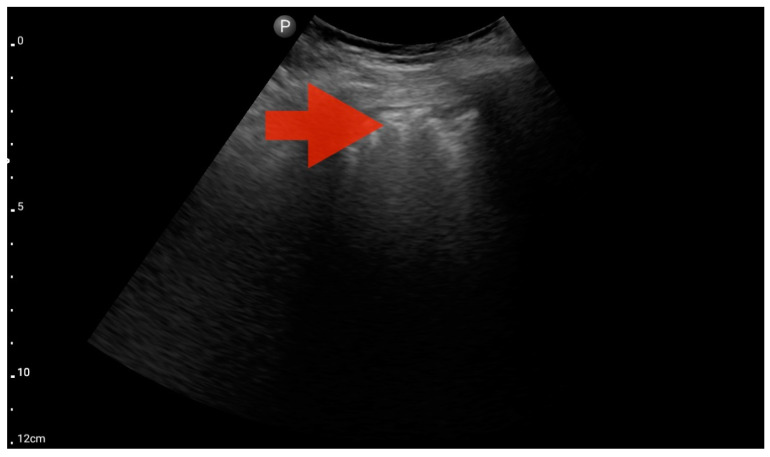
Profile B/C (BLUE protocol). Convex probe examination, the middle part of the lung (5–7th intercostal); the red arrow indicates numerous subpleural consolidations with a vertical line—line C (source: author’s material—DK).

In the assessment according to the BLUE protocol, profile B (Figure 7) was observed in seven (43.75%) patients, profile C (Figure 8) in three (18.75%), and profile B/C (Figure 9) in two (12.5%) patients. Profile A was not detected in the study group. Pleural effusion (Figure 10) was the sole observation in four patients. During the assessment according to the eFAST protocol, no additional features beyond the observed information were found in the study group. Such an assessment seemed to be sufficient in 11 (68.75%) patients.

### 3.4. The Assessment According to the FATE Protocol

**Figure 11:**
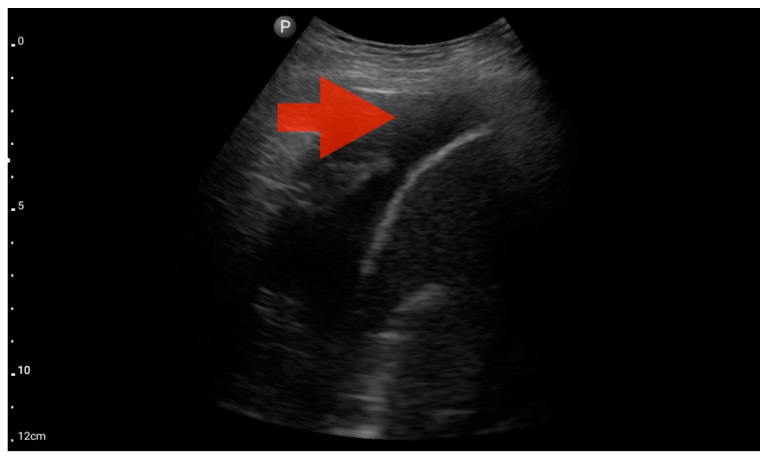
Pleural effusion (BLUE protocol). Convex probe examination, the basal part of the lung (pleural recess); the red arrow indicates free fluid in the pleural cavity (source: author’s material—DK).

Left ventricle hypokinesis, identified through the FATE protocol (Figure 11), was deemed the most valuable information. This observation exhibited a significant association with the hospital-established diagnosis of decompensated heart failure as the primary cause of patient-reported dyspnea. The sensitivity and specificity were 1.0 (95%CI: 0.62–1.0) and 0.6 (95%CI: 0.15–0.95), respectively. The positive predictive value was 0.85 (95%CI: 0.55–0.98), and the diagnostic accuracy was 0.86 (95%CI: 0.62–0.98).

**Figure 12:**
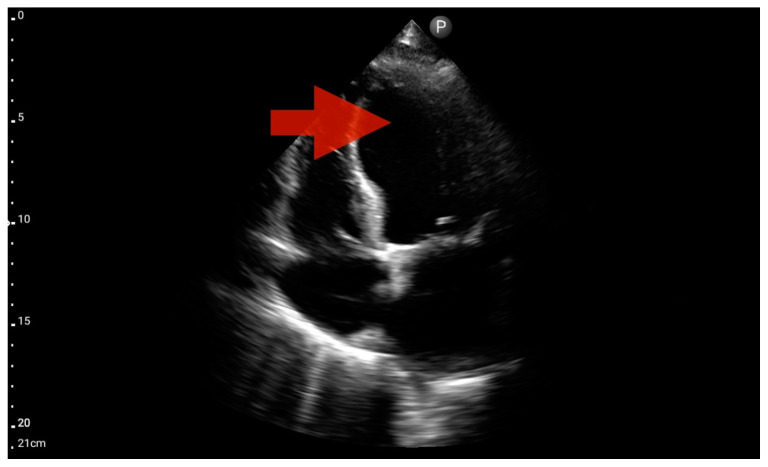
Left ventricle hypokinesis; position: apical four-chamber (FATE protocol). Sector probe; the red arrow marks the left ventricle, which, during the examination, showed signs of reduced ejection fraction and hypokinesis (source: author’s material—DK).

**Figure 13:**
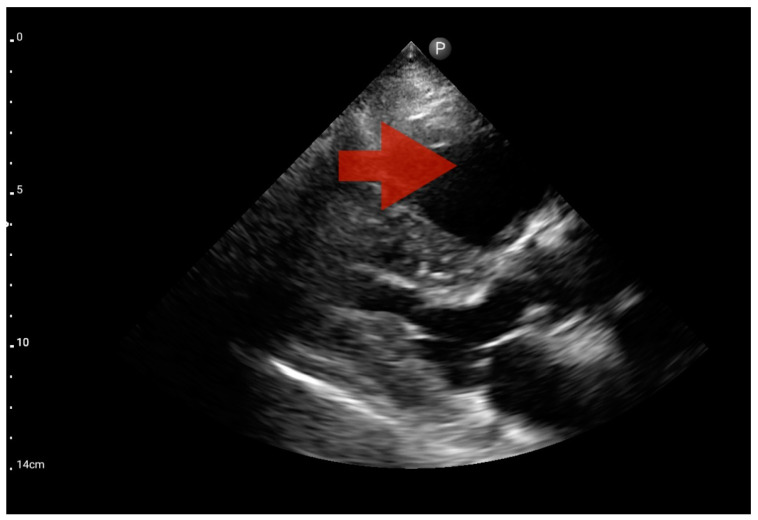
Right ventricle enlargement; position: parasternal long axis (FATE protocol). Sector probe; the right ventricle is marked with the red arrow, and a significant enlargement of the right ventricle is visible, with a shift of the interventricular septum towards the left ventricle, and an ultrasound picture suggesting pulmonary embolism (source: author’s material—DK).

**Figure 14:**
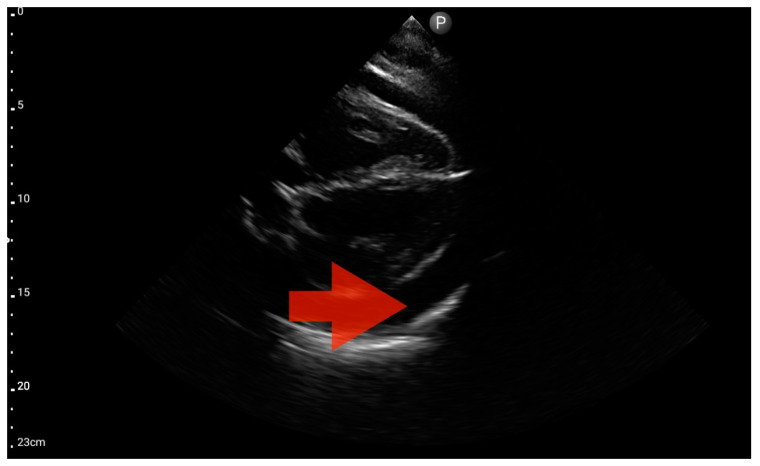
Pericardial effusion; position: apical four-chamber (FATE protocol). Sector probe; the red arrow indicates free fluid in the pericardium, which did not cause a sonographic image of cardiac tamponade (source: author’s material—DK).

Other detected pathologies, including left ventricle enlargement (2 patients; 12.5%) (Figure 12), right ventricle enlargement (2 patients; 12.5%) (Figure 13) and pericardial effusion (1 patient; 6.25%) (Figure 14), were observed only incidentally. Therefore, further validation in a larger-scale study is warranted to analyze the significance of these symptoms in the pre-hospital setting.

**Figure 15:**
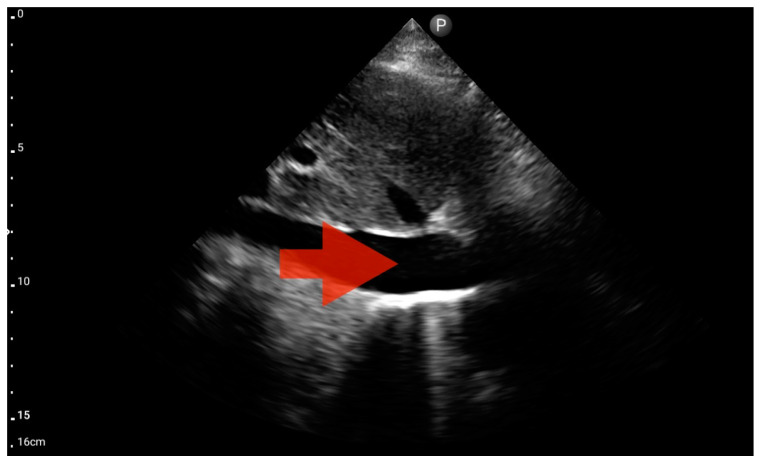
Inferior vena cava (IVC) dilation. Sector probe; the red arrow marks the inferior vena cava; the width is not dependent on the phase of the patient’s breath and shows significant dilatation (source: author’s material—DK).

In our study, the FATE protocol was also followed by the assessment of the inferior vena cava (IVC) diameter. IVC dilation was observed in three patients (Figure 15). We did not find this parameter useful in our pre-hospital setting. Furthermore, such assessment is relatively challenging and not always possible to perform quickly. In four of the analyzed patients, IVC assessment was not possible. As a result, in our group, the sensitivity and specificity for the IVC assessment were 0.29 (95%CI: 0.04–0.71) and 0.8 (95%CI: 0.28–1.0), respectively. The positive predictive value was 0.67 (95%CI: 0.09–0.99), and diagnostic accuracy was 0.5 (95%CI: 0.21–0.79).

FATE imaging was deemed pivotal for facilitating the differential diagnosis (pneumonia coexistent with heart failure exacerbation or monolateral pleural effusion in heart failure exacerbation), guiding clinical deliberations, thereby markedly shaping subsequent diagnostic and therapeutic strategies in five patients, while in the remaining cases, it assumed an ancillary yet consequential role.

## 4. Discussion

To summarize the conclusions of this study, we would like to clearly emphasize the benefits of using ultrasound imaging of the heart and lungs as part of pre-hospital care for a patient with respiratory failure. The complexity and issues of differential diagnosis in patients with simultaneous heart and lung disease indicate the need to use targeted and quick diagnostics, which can be done via ultrasound examination.

From the available studies and articles, we were unable to find clear and focused publications on the use of diagnostic ultrasound for the differential diagnosis of dyspnea using cardiac and pulmonary sonography in the pre-hospital setting. There are papers on differential diagnosis using LUS but without myocardial diagnosis [10].

Based on two case reports, the authors presented the use of pre-hospital differential diagnosis of pulmonary edema and COPD using LUS. In both cases, the differential diagnosis involved patients with both pulmonary and cardiac diseases. Ultrasound was used in a patient with respiratory tract obstruction, where, after the LUS examination, a B-profile was identified, and treatment for pulmonary edema was initiated, while in the second case, in a patient with severe heart failure, an A-profile was identified on LUS, and based on further physical examination, airway obstruction was diagnosed, and bronchodilator treatment was initiated with satisfactory results. Zechner’s paper did not address the diagnosis of POCUS ECHO, e.g., FATE, which we believe would be a beneficial adjunct when examining patients with heart failure [10].

Most of the papers available in PUBMED describe cardiac diagnostics in the pre-hospital setting in terms of targeted imaging of the heart or lungs only, usually based on clinical case reports [11]. In his publication, Jakobsen et al. describe two cases of using sonography to identify severe myocardial pathology (pulmonary embolism and pericardial fluid) in the pre-hospital setting, as part of care provided by airborne teams—HEMS. The authors place great emphasis on the fact that the performance of pre-hospital diagnostic cardiac ultrasound allowed them to make decisions about the treatment of these patients and point out that this study guided their decisions regarding the choice of destination hospital, given the needed diagnostic and therapeutic treatment, e.g., for pulmonary embolism. The authors signal the importance of the scope of ultrasound training in the skillful and rapid diagnosis of ultrasound and specify its validity and suitability in the diagnosis of chest pain, dyspnea, and sudden cardiac arrest. The rationale for the use of ultrasound in the diagnosis of free pericardial fluid and the differential diagnosis of traumatic cardiac tamponade was noted and described by William Heega-ard et al. [12].

In a review of the utility of ultrasound in nontraumatic patients in whom ultrasound was performed, a hypothesis was put forward that there is a lack of randomized trials focused on ultrasound diagnosis in the pre-hospital setting [13]. The authors emphasize the effectiveness of ultrasound; however, they point out the heterogeneity and high risk of bias in available studies.

In the existing body of literature, publications corroborate, following a thorough examination of the research, the efficacy and rationale of globally employing ultrasonography (USG) for the diagnosis of critical pathological conditions in pre-hospital contexts, both in the United States and Europe [14]. The deployment of ultrasound in pre-hospital environments is frequently expounded in the context of integrating supplementary training modules for physicians specializing in pre-hospital medicine [15]. The referenced study constitutes a significant voice in our publication, underscoring the intricacies inherent in the differential diagnosis of elderly patients with heart failure. The authors emphasize that the implementation of ultrasound training has contributed to an enhanced diagnostic quality executed by physicians in pre-hospital settings, asserting its pivotal role in a comprehensive evaluation.

The principal limitation of our study is the relatively small number of participants, categorizing it as a pilot study and emphasizing its hypothesis-generating nature. This limitation is rooted in the exigent circumstances wherein patients, frequently presenting with life-threatening conditions, pose challenges in recruiting a larger cohort. The exclusion of unconscious individuals further restricted our target demographic. Difficulties in assembling a sizable cohort were compounded by the integration of POCUS into official medical documentation and the dissemination of findings to hospital personnel, potentially introducing biases. Nonetheless, it is imperative to recognize that executing a randomized, blinded study is ethically and legally impractical.

## 5. Conclusions

Combined ultrasound examination of the heart and lungs in patients with shortness of breath seems to be a valuable diagnostic element when caring for patients with respiratory failure in pre-hospital conditions. The limited scope of possible diagnostics in such conditions makes ultrasound examination comprehensive and accurate in identifying the problems of the underlying disease causing shortness of breath. In conclusion, the integration of the FATE protocol into the BLUE and eFAST procedures appears to be a valuable strategy for improving the accuracy of the pre-hospital differential diagnosis of pleural effusion in adults. The synergistic application of these protocols not only streamlines diagnostic processes but also has the potential to contribute to more informed and efficient clinical decision-making. Further validation in a larger-scale study is warranted to substantiate our findings and enhance the generalizability of the conclusions.

The use of cardiac ultrasound imaging following the FATE protocol in patients with shortness of breath allows for a targeted diagnosis, which enables the implementation of accurate and safe treatment in patients encountered in pre-hospital conditions. An additional aspect is the fact that such an approach allows the exclusion of the cardiac etiology of shortness of breath, which translates into an increase in the quality of care in patients with life-threatening respiratory failure in pre-hospital settings.

We suggest the need for future clinical research into the use of ultrasound for the pre-hospital diagnosis of patients with multiple comorbidities, in particular lung and heart diseases. We believe it is reasonable to develop a targeted heart and lung ultrasound examination protocol in the future that can be used for the differential diagnosis of the causes of shortness of breath in pre-hospital conditions.

## Figures and Tables

**Table 1:** The baseline characteristics of the patients.

Parameter	Result
**Demographics**	
Age, years, median [IQR]	76 [72.5–85.25]
Gender	
Male, *n* (%)	8 (50)
Female, *n* (%)	8 (50)
**Laterality of pleural effusion**	
Monolateral, *n* (%)	5 (31.25)
Bilateral, *n* (%)	11 (68.75)
**Primary hospital-acquired diagnosis**	
Decompensated heart failure, *n* (%)	11 (68.75)
Pneumonia, *n* (%)	4 (25)
Pulmonary embolism, *n* (%)	1 (6.25)
Chronic kidney disease, *n* (%)	1 (6.25)
Lung cancer, *n* (%)	1 (6.25)

**Table 2:** Comparison of the use of combined heart (FATE) and lung (BLUE/eFAST) ultrasound diagnostics in the context of separate lung and heart diagnostics.

Patient Number	USG—BLUE/eFAST Protocol (Diagnosis Based on Lung Examination)	USG—FATE Protocol (Diagnosis Based on Heart Examination)	Final Diagnosis (Diagnosis Based on Combined Heart and Lung Examination)
1.	Pulmonary oedema	LV heart failure	LV heart failure
2.	Pleural effusion	Heart failure	Heart failure
3.	Pneumonia	No imaging changes	Pneumonia
4.	Bronchial asthma	No imaging changes	Bronchial asthma
5.	Pulmonary oedema	LV heart failure	LV heart failure
6.	Pneumonia	Heart failure	Pneumonia
7.	Pneumonia	LV heart failure	Pneumonia
8.	Pneumonia	No imaging changes	Pneumonia
9.	Pulmonary oedema	Heart failure	Heart failure
10.	Pleural effusion	Heart failure	Heart failure
11.	Pleural effusion	Heart failure	Heart failure
12.	No imaging changes	Pulmonary embolism	Pulmonary embolism
13.	Pulmonary oedema	LV heart failure	LV heart failure
14.	Pneumonia	Heart failure	Pneumonia
15.	Pulmonary oedema	Heart failure	Heart failure
16.	Pneumonia	No imaging changes	Pneumonia
17.	Pneumonia	No imaging changes	Pneumonia
18.	Pleural effusion	Heart failure	Heart failure
19.	Pneumonia	Heart failure	LV heart failure
20.	Pneumonia	No imaging changes	Pneumonia
21.	Pulmonary edema	LV heart failure	LV heart failure
22.	Pulmonary edema	LV heart failure	LV heart failure
23.	Pleural effusion	No imaging changes	Lung cancer
24.	Pulmonary edema	Heart failure	LV heart failure
25.	Pulmonary edema	Heart failure	Heart failure

**Table 3:** Comparison of diagnoses by three investigators based on the assessment of the same sonographic images.

Patient Number	Diagnosis Based on the BLUE/eFAST Protocol: DK/AB/MT	Diagnosis Based on the FATE Protocol: DK/AB/MT	Final Diagnosis Based on the BLUE/eFAST and FATE Protocols: DK/AB/MT
1.	Pulmonary edema/pulmonary edema/pulmonary edema	LV heart failure/LV heart failure/LV heart failure	LV heart failure/LV heart failure/LV heart failure
2.	Pleural effusion/pleural effusion/pleural effusion	Heart failure/heart failure/heart failure	Heart failure/heart failure/heart failure
3.	Pneumonia/pneumonia/pneumonia	No imaging changes/no imaging changes/no imaging changes	Pneumonia/pneumonia/pneumonia
4.	Bronchial asthma/bronchial asthma/bronchial asthma	No imaging changes/no imaging changes/heart failure	Bronchial asthma/bronchial asthma/bronchial asthma
5.	Pulmonary edema/pulmonary oedema/pulmonary oedema	LV heart failure/LV heart failure/LV heart failure	LV heart failure/LV heart failure/LV heart failure
6.	Pneumonia/pneumonia/heart failure	Heart failure/heart failure/heart failure	Pneumonia/pneumonia/heart failure
7.	Pneumonia/pneumonia/heart failure	LV heart failure/LV heart failure/LV heart failure	Pneumonia/pneumonia/pneumonia
8.	Pneumonia/pneumonia/pneumonia	No imaging changes/heart failure/no imaging changes	Pneumonia/pneumonia/pneumonia
9.	Pulmonary edema/pulmonary edema/pulmonary oedema	Heart failure/heart failure/heart failure	Heart failure/heart failure/heart failure
10.	Pleural effusion/pleural effusion/pleural effusion	Heart failure/heart failure/heart failure	Heart failure/heart failure/heart failure
11.	Pleural effusion/pleural effusion/pleural effusion	Heart failure/heart failure/heart failure	Heart failure/heart failure/heart failure
12.	No imaging changes/no imaging changes/no imaging changes	Pulmonary embolism/pulmonary embolism/heart failure	Pulmonary embolism/pulmonary embolism/pulmonary embolism
13.	Pulmonary edema/pulmonary edema/pulmonary edema	LV heart failure/LV heart failure/LV heart failure	LV heart failure/LV heart failure/LV heart failure
14.	Pneumonia/pulmonary edema/pulmonary edema	Heart failure/heart failure/heart failure	Pneumonia/heart failure/heart failure
15.	Pulmonary edema/pulmonary edema/pneumonia	Heart failure/heart failure/heart failure	Heart failure/heart failure/pneumonia
16.	Pneumonia/pneumonia/pneumonia	No imaging changes/no imaging changes/heart failure	Pneumonia/pneumonia/pneumonia
17.	Pneumonia/pneumonia/heart failure	No imaging changes/no imaging changes/no imaging changes	Pneumonia/pneumonia/pneumonia
18.	Pleural effusion/pleural effusion/pleural effusion	Heart failure/heart failure/heart failure	Heart failure/heart failure/heart failure
19.	Pneumonia/pneumonia/pneumonia	Heart failure/heart failure/heart failure	LV heart failure/LV heart failure/LV heart failure
20.	Pneumonia/pneumonia/pneumonia	No imaging changes/no imaging changes/no imaging changes	Pneumonia/pneumonia/pneumonia
21.	Pulmonary edema/pulmonary edema/pulmonary edema	LV heart failure/LV heart failure/LV heart failure	LV heart failure/LV heart failure/LV heart failure
22.	Pulmonary edema/pulmonary edema/pulmonary edema	LV heart failure/LV heart failure/LV heart failure	LV heart failure/LV heart failure/LV heart failure
23.	Pleural effusion/pleural effusion/pleural effusion	No imaging changes/no imaging changes/heart failure	Lung cancer/pleural effusion/lung cancer
24.	Pulmonary edema/pulmonary edema/pulmonary edema	Heart failure/heart failure/heart failure	LV heart failure/LV heart failure/LV heart failure
25.	Pulmonary edema/pneumonia/pulmonary edema	Heart failure/heart failure/heart failure	Heart failure/heart failure/heart failure

## Data Availability

The data utilized and/or analyzed in this study can be made available by the corresponding author upon reasonable request.
